# An Optical Tomography-Based Score to Assess Pediatric Hand Burns

**DOI:** 10.3390/ebj5020013

**Published:** 2024-05-15

**Authors:** Judith Lindert, Tina Straube, Beke Larsen, Julia Siebert, Eirini Liodaki, Kianusch Tafazzoli-Lari, Lutz Wünsch

**Affiliations:** 1 Department of Pediatric Surgery, University Hospital Lübeck, Ratzeburger Alle 160, 23538 Lübeck, Germany; tina.straube@uk-halle.de (T.S.); bekesophie.larsen@uksh.de (B.L.); julia.siebert@uksh.de (J.S.); kianusch.tafazzoli-lari@uksh.de (K.T.-L.); 2 Department of Pediatric Surgery, University Hospital Rostock, Ernst-Heydemann Str 8, 18057 Rostock, Germany; 3 Department of Pediatric Surgery, University Hospital Halle, Ernst-Grube-Straße 40, 06097 Halle (Saale), Germany; 4 Department of Plastic Surgery, University Hospital Lübeck, Ratzeburger Alle 160, 23538 Lübeck, Germany; liodaki_irene@yahoo.com

**Keywords:** burn wound assessment, burn depth assessment, optical coherence tomography, pediatric burn, hand burn, OCT burn score, imaging of burns

## Abstract

To define the morphologic pattern of pediatric hand burns as visualized via optical coherence tomography (OCT) and dynamic OCT (D-OCT). We designed a scoring system to assess the depths of burn wounds on pediatric hands and tested this score in our cohort of children with burn injuries to the hand. Overall, 67 hand burns in 48 children (0–15 years) were prospectively examined. Scans were interpreted by two independent observers. Relevant OCT findings were surface irregularity, loss of epidermis, loss of dermal pattern (skin lines or papillary spots, loss of surface regularity and irregular vascular pattern of the plexus papillaris. Score values were calculated retrospectively. A score of 4 was associated with spontaneous healing without the need for skin grafting, with a positive predictive value of 97%. Deeper wounds with delayed healing and/or the need of skin grafting received a score of 5 or above, with an agreement of medical healing in 80% and a positive predictive value of 56%. OCT and D-OCT provide clinically useful additional information in cases of pediatric hand burns. The OCT burn score has the potential to support clinical decision making and, subsequently, improve clinical outcomes and shorten hospital stays.

## 1. Introduction

Hand burns are among the most serious injuries in childhood and may have an enormous impact on a child’s future development. The German Burns Registry recorded 2595 pediatric burns in 2021, of which 58% were in infants under 1 year of age and 30% involved the hand [1]. Contact burns from ovens or hot plates are very common: children often fail to withdraw their hand immediately, resulting in deep contact burns. Generally, the skin of children is thinner compared to adults. The back of the hand is more commonly involved in scalds, and often combined with other body areas [2]. Although hand injuries are not life-threatening, they are relevant because palms and fingers are highly innervated, and burns are very painful. The functional structures are very superficial and easily injured, resulting in limited motion ranges of the hand and fingers. Scarring can lead to functional and cosmetic impairments [2,3]. Appropriate treatment is essential, and special expertise is required to decide when and how to use skin grafts. Superficial wounds heal within two weeks with no long-term sequelae [3]. Deep wounds require skin grafts, and scarring is expected. Even with optimal treatment, the results of skin grafts are far from ideal because the structure of the skin is irreversibly altered by debridement and structural changes during the healing process. Graft contraction is common and may lead to secondary procedures. Accurate assessment of wound depth—even in the hands of highly experienced clinicians—remains challenging, as none of the clinical features used to assess burn depth appear to be reliable predictors of wound healing potential [4,5]. Therefore, choosing the ideal time for skin grafting remains a dilemma; grafting too early may sacrifice wound areas that would otherwise heal, and/or grafting too late may prolong hospital stay and increase wound infection rates [4,5].

The degree of dermal loss and damage to the vascular plexus of the skin define the potential for spontaneous wound healing and, thus, the severity of the burn injury and should be considered in the assessment of the burn depth [2,4]. 

Our research aims to improve early burn wound assessments, allowing for early definitive wound care. Several imaging techniques are currently being investigated in this area, such as Laser Doppler Imaging, which generates a color-coded image of the perfusion. The method has shown its ability to detect different burn depths. LDI does not provide any structural information about the wound [6,7]. Other non-invasive optical techniques for wound imaging include hyperspectral imaging, multispectral imaging, near-infrared spectroscopy (NIRS), and diffuse reflectance spectroscopy. These techniques visualize the oxygenation of the wound and reflect cutaneous oxygenation [8,9].

Our study is based on optical coherence tomography (OCT), a technique that provides both morphological images of the skin and the blood flow to the papillary plexus of the skin.

We obtained OCT and dynamic OCT (D-OCT) images of pediatric hand burns. We derived four morphologic criteria to assess the burn depth (surface irregularity, loss of epidermis, either papillary pattern in thin skin (hairy) or skin lines in thick skin (glabrous), and microvascular network) that we identified in our pilot study [8]. These criteria were used to develop a clinical score.

We evaluated OCT scans of the palm and back of the hand to address three research questions:

(1) Could optical coherence tomography be used to differentiate thermal injury from healthy skin in children with hand burns?
(2) Is it possible to differentiate deep wounds eventually requiring skin grafts from superficial wounds that would heal spontaneously as early as 48 h after injury?
(3) Can OCT patterns of hand burns be summarized into a clinically useful score?

## 2. Materials and Methods

To address these three questions, we designed a prospective longitudinal observational study. The study protocol was approved by the institutional review board (15–116 on 10.06.2015) of the University of Lübeck.

Children admitted to the pediatric burn center at the University Medical Center Schleswig-Holstein (UKSH), Campus Lübeck, Germany, between January 2016 and December 2021 were eligible.

Altogether, 461 children with thermal injuries were admitted during the study period, of which 171 (37.1%) had hand injuries. OCT was integrated into the wound care protocol on the second day after injury. OCT images were available for 69 patients with injuries to the hand. After an assessment of image quality, we could include images of 67 hands from 48 patients with a complete dataset for analysis.

Wound care included debridement, clinical evaluation, and OCT scanning. Wounds were dressed with an alloplastic epidermal substitute (Suprathel^®^-PolyMedics Innovations GmbH, Denkendorf, Germany) [10].

Wound morphology and scan position were documented with a digital photograph. A similar area of healthy skin, either adjacent or contralateral, was scanned for comparison. The OCT scans were performed by an additional person in order to save time and limit the anaesthesia time. We included data on patient characteristics, admission patterns (direct and referral), injury characteristics (side of injury, surface, cause of injury, and injury pattern), number of dressing changes, and need for skin grafting.

Data on patient demographics and injury-related characteristics were retrieved from patient charts, pseudonymized, and entered into a database using Excel.

Descriptive statistical analysis was carried out by J.L., T.S., J.S. using SPSS 25.0.

### 2.1. OCT and D-OCT

The Vivosight Dx© OCT device (Michelson Diagnostics Ltd., Kent, UK) was used for this study. The Vivosight Dx© OCT device is licensed for clinical use and provides simultaneous OCT and D-OCT images with a scan time of 30 s per scan consisting of 120 consecutive images. It is a swept-source Fourier-domain OCT based on multi-beam technology and provides a resolution of <6 microns axially and <7.5 microns laterally. Each scan visualizes a 6 mm × 6 mm area of skin. It produces cross-sectional images of the skin with a very high resolution of 3–5 μm, and the depth of penetration is 2 mm. A three-dimensional reconstruction, the “enface” view, can be derived from the serial reconstruction of 120 sectional images [7,10,11]. Movement artifacts can occur, and the stabilization of the scanner–wound distance was achieved using a disposable plastic spacer.

To create an OCT image, low-coherence interferometry produces a cross-sectional image through optical scattering of a laser beam, providing structural information about the skin and creating a visual image [7,12,13,14,15].

The OCT images were examined in both longitudinal sections and en-face views, which are mandatory for the interpretation of vascular patterns in dynamic OCT (D-OCT).

D-OCT provides images of the vascular anatomy based on the speckle variance of repeated OCT scans by recording the signal changes of moving erythrocytes in the blood flow [15]. Subsequently, visualization of the microvascular plexus is possible. The signals for OCT and D-OCT are collected simultaneously during the same scan [11,12,13].

For the first study question, two physicians (JL and LW) independently analyzed the OCT and D-OCT images of the hand wounds and a matched site using the already identified burn injury patterns visualized via OCT [8].

After an initial descriptive data analysis, a score was developed and retrospectively assigned. The score is based on the characteristic wound criteria defined and visualized via OCT in our pilot study [8]:

(1) Surface irregularity,
(2) Loss of epidermis,
(3) Dermal pattern: skin lines or papillary pattern
(4) Perfusion/microvascular network.

### 2.2. Score Design

To each feature, a value was assigned. Surface irregularity and epidermal loss each receive a maximum of one point. Dermal patterns and vascularity were scored from 0 to 2 (Table 1). The score was 0 for healthy skin. The maximum score of 6 would indicate a deep burn with a destroyed dermal structure and absent dermal plexus.

Figures 2–4 illustrate different wounds visualized using OCT, ranging from normal skin to deep burns; en-face views of dermal injuries of glabrous and hairy skin are listed separately. See the proposed score in Table 1.

In the second step, we applied our proposed score to the OCT images of hand burns. We assessed the initial clinical wound judgement, the medical healing and the assigned score of the images taken on day 2 after injury.

## 3. Results

From January 2016 to December 2021, 461 children with burns were admitted, 171 (37.1%) sustained an injury to their hand. The ages of these patients ranged from 3 months to 15 years; 113 were male, and 58 were female. The mean length of hospital stay was 6.1 days (SD 6.6). Most patients required 2.9 (SD 1.9) dressing changes. If a skin graft was needed, it was performed after 9 days (median). Patient characteristics are described in Table 2.

OCT scans from 69 patients were eligible and included in our study. Datasets of 52 patients with 66 hand injuries were of good image quality and had complete labeling. We excluded images when the image quality was insufficient for analysis due to artifacts. For an overview of wound characteristics and OCT features, see Annex/short summary visualized in Table 3.

### 3.1. Epidermis

**Figure 1:**
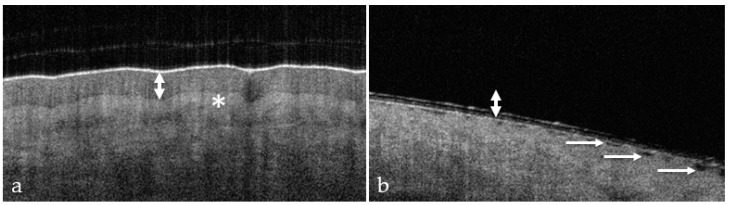
OCT visualization of the epidermis in healthy skin (**a**) and thermally injured skin (**b**): epidermis/loss of epidermis (double arrow), DEJ (star), and surface irregularity (simple arrows).

OCT of uninjured skin showed the epidermis as a dark band (Figure 1a) with a relatively smooth surface, and fine wrinkles were observed in areas of very pliable skin overlying the articulations. The dermo–epidermal junction was marked by a visible step from dark grey (epidermis) to light grey (dermis).

Surface irregularity (Figure 1) is a characteristic, universal feature of all wounds and was present in each wound. Blistering occurs near the dermo–epidermal junction (DEJ) and causes the disappearance of the dark band in the OCT image (Figure 1b). Both signs are easily identified, and each results in a score of 1.

**Figure 2:**
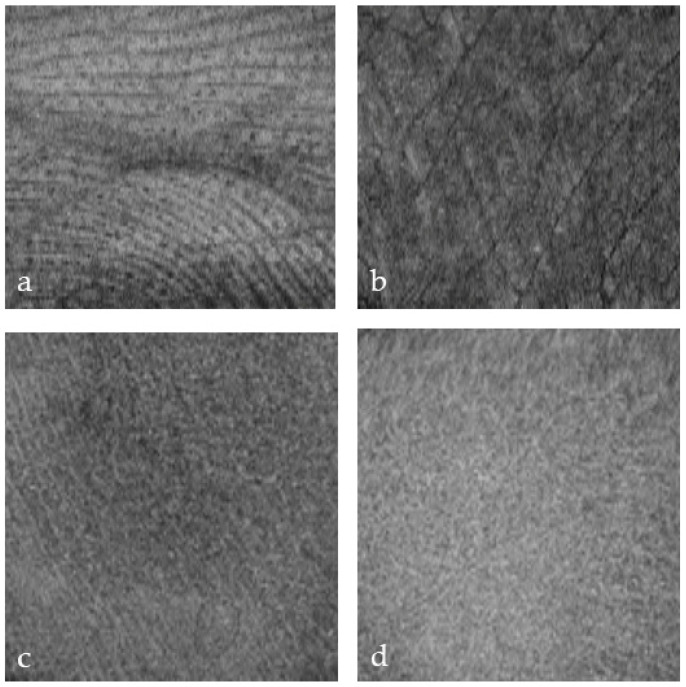
OCT of glabrous skin at different depths: (**a**) 0.3 mm, (**b**) 0.6 mm, (**c**) superficial burn 0.3 mm, and (**d**) deep burn.

Glabrous skin has a thicker epidermis and sweat glands with visible ducts. The characteristic skin lines are visualized (Figure 2a,b) from the en-face perspective. The skin covering the dorsum of the hand has a thinner epidermal band. Skin thickness varies in different areas of the body and with age (Figure 2).

### 3.2. Dermis

The dermo–epidermal junction (DEJ) is characterized by a fine line and transitions to a brighter shade in the longitudinal view. This result was due to the high collagen content of the reticular dermis (Figure 1).

**Figure 3:**
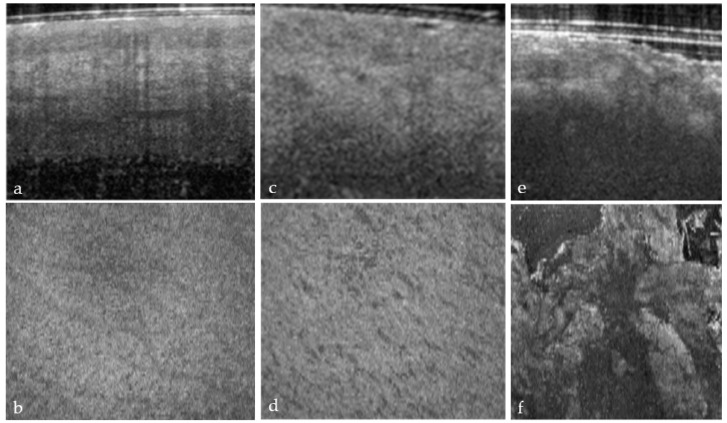
Hair-bearing skin papillary pattern. (**a**) Healthy skin, (**b**) healthy skin en-face view, (**c**) superficial burn, (**d**) superficial burn en-face view partially damaged, (**e**) deep burn, and (**f**) deep burn en-face view totally disrupted papillary pattern.

Skin lines are a characteristic feature of the glabrous skin of the palms and the soles (Figure 2). OCT detected skin lines in burn wounds from the en-face perspective, even when the lines were clinically invisible. Skin lines can be visualized in the dermis at depth (Figure 2a,b). We observed that grafting became necessary when the skin lines disappeared, indicating a deep dermal injury. (Figure 2d). More importantly, persistent lines (Figure 2c) were associated with spontaneous healing and may thus represent a favorable outcome marker. In superficial palm burns, the skin lines become blurred at the 0.3 mm level (Figure 3c) and disappear completely in deep burns (Figure 2d).

The en-face view of hair-bearing skin displays a characteristic pattern of small, dark spots at the level of the papillary dermis (Figure 3b). These spots represent openings in the dermal matrix for hair and the papillary vascular plexus. These spots are regularly spaced in the papillary dermis (Figure 3b), and the loss of this pattern (Figure 3f) indicated a deeper dermal wound that would eventually require a skin graft. We only observed this pattern in hear-bearing skin. The partial (Figure 3d) or complete disruption (Figure 3f) of this pattern was observed, and a score of 1 or 2 was assigned.

### 3.3. Vascular Structures

In healthy skin, the en-face view showed an organized microvascular network, and individual capillaries could be identified.

If the superficial vascular plexus is damaged, the wound is at risk of poor healing [15].

**Figure 4:**
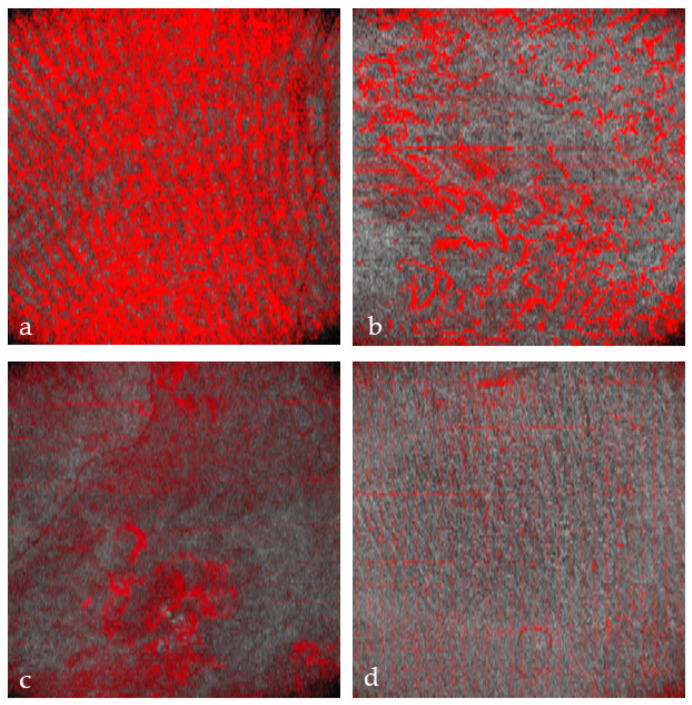
Dynamic OCT image visualizing vascular network (all en-face views). (**a**) Healthy skin with intact vascular plexus, (**b**) superficial burn with partially disrupted vascular network, (**c**) deep burn that is totally disrupted, truncated vessels, and (**d**) deep burn with absent vascular plexus.

Thus, D-OCT provides images of the superficial vascular plexus. In healthy skin, en-face views show a fine vascular network with decreasing vessel size at each point of arborization (Figure 4a).

Burn injuries may damage the vascular architecture, and the reticular structure may disappear partially (1 point; Figure 4b) or completely (2 points; Figure 4c, d). First, small vessels disappear (Figure 4b), leaving only relatively thick vessels without branches (Figure 4c).

The preservation of the vascular pattern in our patients, as visualized in Figure 4b, was associated with spontaneous wound healing.

### 3.4. OCT Burn Score

The scores were computed in retrospect to provide a quantitative description of the burn severity, as follows (Table 3):

Score 0–2: Healthy skin or epidermal injury;

Score 3–4: Partially thickness burn with spontaneous healing;

Score 5–6: Full-thickness burn needing grafting.

We assigned a score of 2 twice, a Score of 3 fifteen times, a score of 4 twenty-nine times, a score of 5 twelve times and a score value of 6 five times. The overall characteristics and OCT scores are displayed in Table 3.

All wounds with a score of 4 or less healed spontaneously with wound dressing. Wounds with a score of 5 or more proved to be deeper, and the majority needed skin grafting.

The initial clinical judgment assigned a deeper appearance to 11 hands (0.16%) and the wounds healed spontaneously. In one hand, the initial clinical judgment was too superficial (1.5%). The agreement between medical healing and a score of 4 or below was 97.8%, with a positive predictive value for healing of 97%. It seems that the initial clinical judgment may rate wounds too deep.

The agreement of a deep wound on medical healing and a score of 5 or more was 80%, and 9 of those 15 hands (53%) with a score of 5 or more underwent skin grafting after a median of 9 days. The positive predictive value for skin grafting when the score was 5 or more is 56%. One patient had a clinically deep appearance, deep appearance at the time of medical healing, and an OCT Score of 6 and bilateral injury refused skin grafting with the subsequent development of a hypertrophic scar.

Thus, a clinically valuable threshold could be defined by the combination of dermal and vascular injury patterns.

## 4. Discussion

Our study provides a comprehensive description of OCT as an imaging modality in cases of pediatric hand burns. The dataset is large and focuses on a well-defined group.

The clinical and epidemiologic characteristics of our patients reflect those registered in our national registry. Most children were 2 years and younger [1].

In our study, OCT reliably distinguished normal skin from skin with burn injuries [8], answering our first research question. Furthermore, we were able to reliably image the characteristics of deep dermal and vascular injury patterns in pediatric patients with hand burns using OCT. Our previous research identified distinctive OCT features of burned skin compared to healthy skin in a pediatric population [8]. The strength of this technology is the combined detection of skin anatomy and blood flow.

Experimental and clinical evidence based on biopsies clearly point to the relevance of vascular plexus as a prognostic factor [4,5,16,17,18]. Deegan et al. recently summarized the characteristics of different imaging modalities for burn wound assessment and concluded that OCT is the most promising adjunct to clinical judgment because it simultaneously provides information on the morphologic structure and the microperfusion [6,8,17]. Because only a small portion of the wound is examined, OCT is theoretically prone to bias caused by a sampling error. OCT scans should be obtained from the central part of the wound, which is most often the deepest.

Compared to Laser Doppler Imaging, OCT is not affected by the patient’s body shape and surface temperature. LDI provides a two-dimensional color-coded image of blood flow to the skin, and a large area can be scanned in a comparatively short time [6,7,9,19,20,21]. Small, curved surfaces are less well captured. OCT scanning requires more time than Laser Doppler Imaging, and only a limited wound area can be examined in a useful time frame. A superior quality of structural images and imaging of the vascular architecture compensates for these shortcomings. The hands and fingers of children are small and three-dimensional, and OCT is well suited to adapt to the contour of small structures, whereas LDI is less precise. A major strength of OCT is providing a three-dimensional image with high spatial resolution [8,12]. The epidermis, dermis, dermal appendages and vessels, and blood can be visualized. Because LDI can show blood flow to large surface areas very well, OCT and LDI should be considered complementary techniques, with OCT being more useful for hand burns in children.

Answering the second research question, our results suggest that OCT may be helpful in guiding treatment. Scores of 4 and below do not require grafting, and definitive conservative treatment can be initiated after initial debridement and OCT, with a positive predictive value of 97% [15]. An early prognosis of wound healing reassures the family and facilitates treatment, avoiding unnecessary costs and complications during the observation period.

With values above 5 in the proposed score, a wound will heal in 80% of deep wounds. Our score predicts skin grafting in 56%; however, the decision to perform skin grafting is also influenced by the size of the wound and the families’ wishes. Evaluation of the entire wound can help determine which areas require grafting and avoid unnecessary grafting while initiating timely definitive treatment early. This would allow the immediate grafting of the affected area in the future, reducing the risk of scarring and infection.

Our proposed score facilitates standardized, structured assessments and defines a clinically practical threshold for skin grafting, as asked by our third research question. This OCT burn depth score complements other widely used scores to help us as clinicians facilitate diagnosis and enable the best clinical management [22,23]. Combining imagining with Artificial Intelligence bears huge potential in the future for achieving systematic user-independent assessments [24]. Many aspects of burn wounds may be relevant for prognosis, with depth, localization and size being only very rough estimators of a complex biological process. The countless variations in the contour and depth of burns are not well captured in actual classifications, and many actual unknown factors may play a role in wound healing. Machine learning is increasingly good at memorizing details and pattern recognition, and some yet undiscovered relevant prognostic factors of burn injuries may exist. Machine learning relies on high-quality image data, the kind that OCT provides.

Some limitations of our study need to be addressed. Fifteen patients who underwent OCT hand scans were excluded due to poor image quality. Inadequate images resulted from motion artifacts, mostly due to the scanner sliding on the wound. Therefore, we are aware of the fact that the scanning procedure requires technical improvements. In addition, incomplete labeling of images and wound areas occurred initially, making comparisons difficult in the retrospective analysis. As no discordant results were excluded, we consider the existence of selection bias in our results unlikely.

Sampling errors must also be considered when only a small area (6 mm × 6 mm) of the wound is scanned during a scan. The region of interest may be missed. However, this is also true for wound biopsies. Other potential confounding factors are application pressure and skin temperature, the effects of which have not been studied.

The grading of OCT images is potentially affected by inter- and intra-observer variability. In this study, all of the images were graded independently by two experienced observers, and we aim to address inter-observer variability with multiple observers in a separate study. Despite these limitations, OCT has the potential to improve the diagnosis of pediatric burn wounds and offers a way to avoid overtreatment and undertreatment [8,15,18]. Precise, personalized treatment can improve outcomes and reduce the risks and costs of unnecessary treatment. Our findings add to the extensive research that has been conducted in the field of burn wound assessment and will hopefully help to overcome the inaccuracy and time delay of clinical judgment. A prospective evaluation of the score to test its predictive value is underway.

## 5. Conclusions

OCT and D-OCT reliably visualize the morphologic and microvascular patterns of pediatric burn injuries. The OCT images on the second day after injury show patterns that can be summarized in a clinical score. A score of 4 and below predicts the wound’s potential for spontaneous healing, with a positive predictive value of 97%.

Further refinement of imaging and analysis—potentially using AI—is likely to improve diagnostic accuracy, leading to improved outcomes and the avoidance of unnecessary treatment and costs.

- OCT assessment on the second day after injury appears to be predictive of wound healing potential
- Loss of dermal papillary pattern (hairy skin), skin lines (glabrous skin), and disruption of microvascular arborization pattern predict deep dermal injury.
- A practical scoring system is proposed for objective assessment. Scores below 4 were associated with spontaneous healing.

## Figures and Tables

**Table 1:** OCT Score pediatric burns.

OCT Image Characteristics of Burn Injuries	OCT/D-OCT	Area	Score
Surface irregularity	OCT	All skin areas	0 no, smooth linear surface 1 irregular surface
Loss of epidermis	OCT	All skin areas	0 normal 1 dark epidermal band absent
Glabrous skin: Skin lines	OCT	Palms and soles	0 normal 1 partially visible 2 no skin lines visible
Hair-bearing skin: papillary pattern	OCT	Dorsum of the hand	0 normal 1 partially visible 2 completely disrupted
Perfusion/microvascular network	D-OCT	All skin areas	0 normal network 1 minor damage 2 disruptions of network: truncated vessels

**Table 2:** Characteristics of all children with thermic injuries to the hand.

Category	All	Male	Female
Gender	171	113	58
Age Mean in years	3.3 (SD 4.6)	3.5 (SD 4.8)	3.1 (SD 4.2)
Cause of injury	Scald 88 Flame 11 Fat burn 4 Contact burn 54 Explosion 12 Other 2		
Location	only injury to hand 82 one other location 36 two or more other locations 53		
Median N° dressing change	2 (Range 1–15, IQR 1)	2 (Range 1–15)	2,5 (Range 1–15)
Median hospital stay in days	4 (Range 0–45, IQR 5)	4	5

**Table 3:** OCT burn score.

OCT Score Items—Images Characteristics of Burn Injuries			
	0 Normal	1 Present/ Attenuated	2 Absent
Surface irregularity	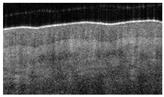	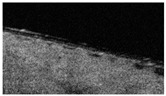	NA
Loss of epidermis	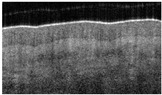	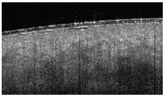	NA
skin lines (glabrous skin)	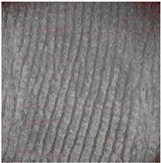	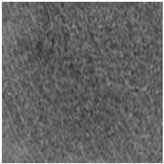	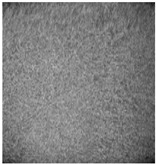
papillary pattern (hair bearing skin)	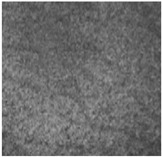	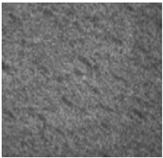	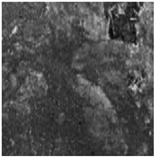
Perfusion/ microvascular network	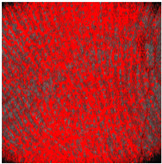	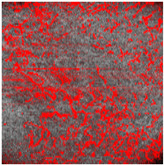	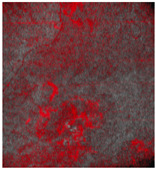

## Data Availability

The data presented in this study are available upon request from the corresponding author. The data are not publicly available due to being sourced from a protected database.

## Abbreviations

MD	Doctor of Medicine
DEJ	Dermo–epidermal junction
D-OCT	Dynamic optical coherence tomography
DTMH	Diploma in Tropical Medicine and Hygiene
IQR	Interquartile range
OCT	Optical coherence tomography
SD	Standard deviation
UK	United Kingdom
UKSH	University Hospital Schleswig-Holstein
